# Epidemiology and treatment of acute elbow dislocations: current concept based on primary surgical ligament repair of unstable simple elbow dislocations

**DOI:** 10.1007/s00068-020-01512-z

**Published:** 2020-10-09

**Authors:** Nils Mühlenfeld, Johannes Frank, Thomas Lustenberger, Ingo Marzi, Anna Lena Sander

**Affiliations:** Department of Trauma, Hand and Reconstructive Surgery, University Hospital Frankfurt, Theodor-Stern-Kai 7, 60590 Frankfurt am Main, Germany

**Keywords:** Acute elbow dislocation, Epidemiology, Treatment

## Abstract

**Purpose:**

Acute elbow dislocations are complex injuries that predispose to chronic instability and pain. The ideal treatment strategy is part of controversial discussion and evidence-based recommendations for the treatment could not be concluded from the literature. The purpose of the present study was to assess current epidemiological data, injury pattern, and the changing trend for treatment.

**Methods:**

This study presents a retrospective review of 72 patients ≥ 18 years of age who were treated in our level I trauma centre with acute elbow dislocations from 2014 to 2018. The data were acquired by analysis of the institution’s database, and radiological examinations.

**Results:**

The average age of the patients was 48.5 years (range 18–86). The ratio of male to female patients was 1.9:1. A fall onto the outstretched arm (42%) was the most common injury mechanism. By classification, 42% of the elbow dislocations were simple, and 58% complex. A total of 85% of patients underwent surgery including 73% of the simple elbow dislocations due to remaining instability or non-congruency of the reduced elbow. The indication for surgical treatment correlated merely with the grade of instability and displacement, but not with age.

**Conclusion:**

Acute elbow dislocations need identification of the precise injury pattern and instability after reduction of the elbow joint. To achieve a congruent and stable joint, we recommend primary surgical repair as first-line treatment for patients with unstable simple and complex elbow dislocation independent of age.

## Introduction

The treatment of acute elbow dislocations is a challenge due the complex interaction between the bony articulations of the elbow joint, the capsuloligamentous structures, and dynamic muscle restraints [1]. Comprehension of the elbow anatomy and the relative contribution of the various elements to elbow stability is important in developing an algorithm for diagnosis and treatment [1]. Additionally, early recognition of the precise injury pattern is critical in restoring elbow function and preventing chronic instability and pain [2]. Despite improvement in understanding of these lesions and a recent increasing trend for early surgical ligament repair, evidence-based recommendations for the treatment could not be concluded from literature [3]. This study was performed to better characterise the current epidemiology, injury pattern, and the trend for surgical treatment of these injuries as important step towards definition of a robust treatment algorithm.

## Patients and methods

**Fig. 1:**
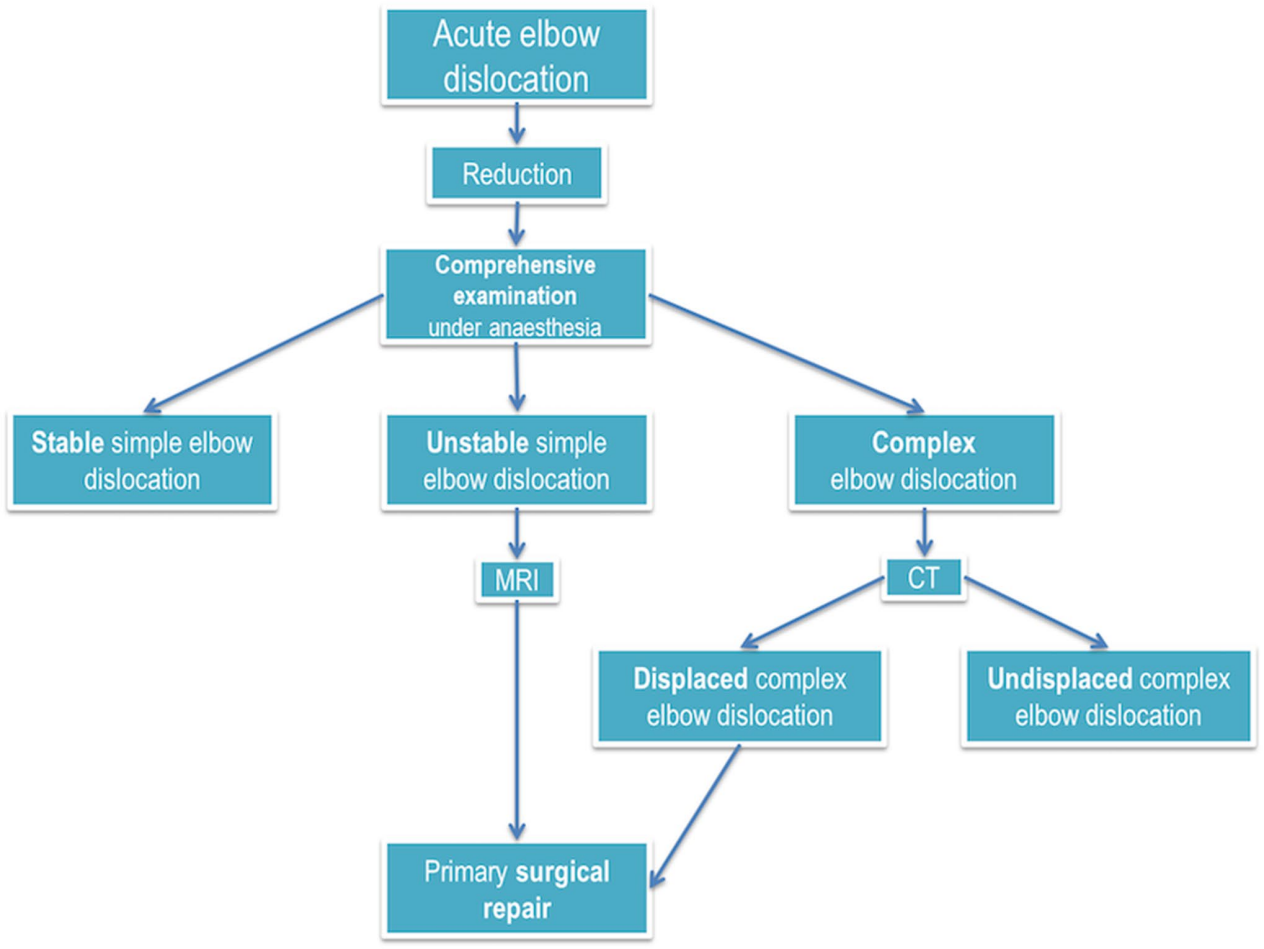
Treatment algorithm for acute elbow dislocations

Approval from the institutional review board of the medical faculty (GN19-390) was obtained prior to performing this retrospective study. The study included all patients ≥ 18 years of age with acute elbow dislocations who were treated in our level I trauma centre according our treatment algorithm between 2014 and 2018 (Fig. 1).

The data were collected by analysis of the institution’s database, and radiological examinations. Information obtained included age, gender, injury mechanism, injury pattern, and mode of treatment. Concerning the injury mechanism, low-energy trauma (i.e., fall from standing or seating height), and high-energy trauma (i.e., fall from height greater than standing, motor vehicle accident, sport accident, bicycle accident) were distinguished.

Elbow dislocations were classified into simple and complex. Simple elbow dislocation occurred without significant associated fractures, while complex elbow dislocations were combined with concomitant periarticular fracture(s) [3–7].

Elbows were usually reduced in the emergency department under analgesia and conscious sedation. After reduction, a comprehensive examination of the joint stability was performed under anaesthesia. Radiographs were performed after reduction in all cases. Stable and reducible injuries were treated conservatively. The indication for surgical treatment included: (1) radiological subluxated or non-congruent joint after reduction, (2) elbows that required an extension limitation over 30° to 45° to maintain reduction, (3) detected instability under anaesthesia, and (4) displaced complex elbow dislocations. All operative patients were treated by surgeons specialised in orthopaedic trauma care.

In non-operative patients, early mobilisation occurred using a hinged orthosis within 2 weeks after trauma. After operative treatment, a long arm cast was applied for 2 weeks. Afterwards, a hinged orthosis was recommended for 4 weeks. Extension was limited for 3 weeks and gradually increased. Forced supination and pronation were restricted for 6 weeks. Full weight-bearing was allowed after a total period of 12 weeks.

Statistical evaluation was performed using chi-square test and Fisher’s exact test. Values of *p* < 0.05 were considered statistically significant.

## Results

### Age and gender

The average age was 48.5 years (range 18–86) containing 79% (57/72) adult patients (< 65 years), and 21% (15/72) elderly patients (≥ 65 years). The ratio of male to female patients was 1.9:1. The average age of males was 42.6 years (range 20–81), and 59.5 years (range 18–86) for females (Table 1).

**Table 1:** Epidemiological and injury details

Number of patients	72
Age (years)	48.5 (18–86)
Gender (male:female)	1.9:1
Injury mechanism	
Fall	42% (30/72)
Bicycle accident	15% (11/72)
Fall from height	15% (11/72)
Ball sports injury	13% (9/72)
Fall down stairs	7% (5/72)
Motor vehicle accident	6% (4/72)
Snowboard accident	3% (2/72)
Direction of displacement	
Posterior	79% (38/48)
Anterior	10% (5/48)
Medial	8% (4/48)
Lateral	2% (1/48)

### Diagnostic procedures

All patients underwent standard of care imaging that included radiographs in 100% (72/72). Computed tomography (CT) was used in 81% (58/72) for the assessment of complex elbow dislocations to delineate fracture type and assist surgical planning as well as on suspicion of a fracture. Magnetic resonance imaging (MRI) was performed in 21% (15/72) providing further information regarding ligament injury. Of these, 20% (3/15) were simple elbow dislocations, 40% (6/15) unstable simple elbow dislocations, and 40% (6/15) complex elbow dislocations.

### Injury mechanism

The injury was caused by low-energy trauma in 42% (30/72), and by high-energy trauma in 58% (42/72). The most typical injury mechanism was fall (42%, 30/72) followed by bicycle accident, and fall from height each accounting for 15% (11/72) (Table 1).

### Distribution of age versus injury mechanism

Reviewing the relationship between age and injury mechanism, 67% (38/57) of adult patients suffered from high-energy trauma, and 33% (19/57) from low-energy trauma, respectively, 27% (4/15), and 73% (11/15) of elderly patients. The differences were statistically significant (*p* = 0.008). Hence, elderly patients sustained predominantly low-energy trauma, while high-energy trauma occurred primarily in adult patients.

### Injury pattern

The most common direction of displacement was posterior (79%, 38/48), thereof posterolateral accounted for 71% (27/38) of the cases, and posteromedial for 29% (11/38). In 33% (24/72) of the cases, the direction could not be determined as reduction was performed prior to hospital admission (Table 1).

**Fig. 2:**
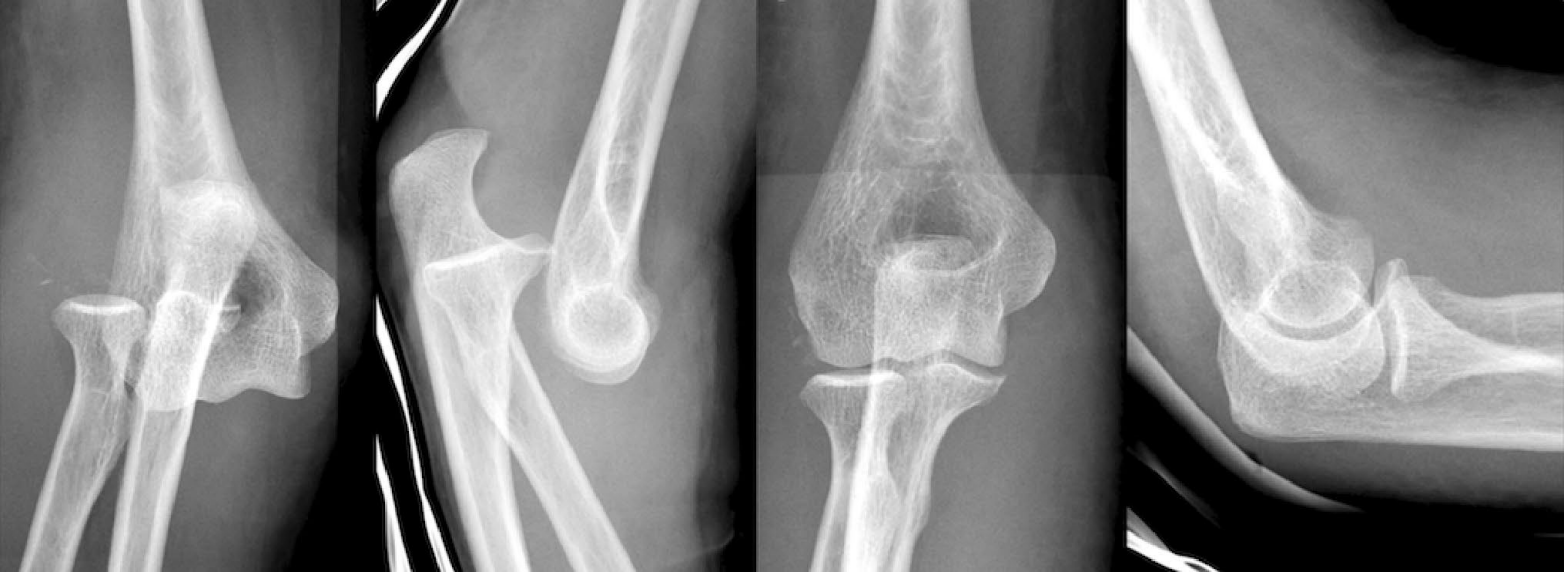
Antero-posterior and lateral radiographs of simple elbow dislocation. **a**, **b** Posterior elbow dislocation. **c**, **d** After closed reduction

**Fig. 3:**
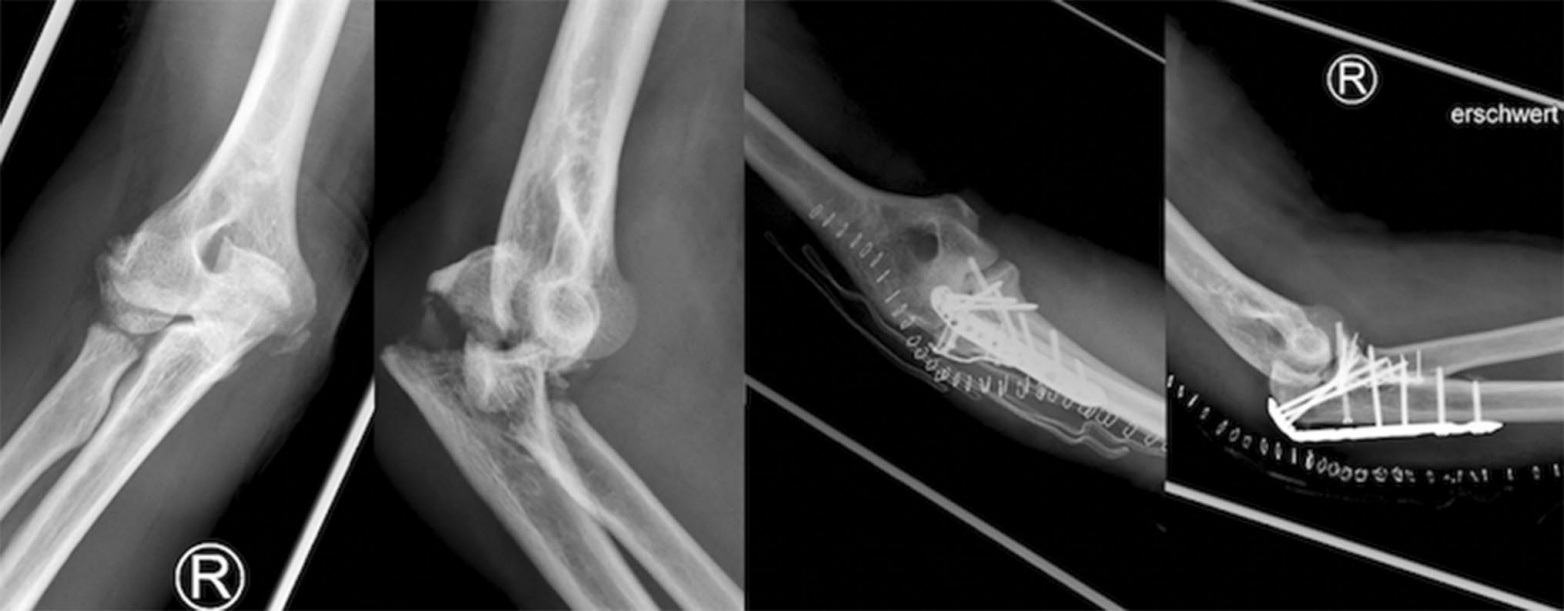
Antero-posterior and lateral radiographs of complex elbow dislocation. **a**, **b** Concomitant fractures. **c**, **d** Internal fixation with locking plates and screws

Simple elbow dislocations without significant associated fractures were seen in 42% (30/72), and complex elbow dislocations with relevant associated periarticular fracture(s) in 58% (42/72) (Figs. 2, 3).

Fractures of the radial head and ulnar coronoid process (33%, 14/42) were the most frequent fracture types, thereof 57% (8/14) “terrible triad” injuries with disruption of the medial collateral ligament, followed by fractures of the ulnar coronoid process (19%, 8/42), and fractures of the radial head (17%, 7/42) (Table 2).

**Table 2:** Distribution of fracture types in complex elbow dislocations

Radial head, ulnar coronoid process	33% (14/42)
Ulnar coronoid process	19% (8/42)
Radial head	17% (7/42)
Radial head, ulnar coronoid process, olecranon/proximal ulna	12% (5/42)
Radial head, olecranon/proximal ulna	10% (4/42)
Radial head, ulnar coronoid process, lateral epicondyle	2% (1/42)
Radial head, capitulum humeri	2% (1/42)
Olecranon	2% (1/42)
Medial epicondyle	2% (1/42)

### Distribution of age versus injury pattern

Evaluating the distribution of age versus injury pattern, 42% (24/57) of adult patients sustained simple elbow dislocations, and 58% (33/57) complex elbow dislocation, versus 40% (6/15), and 60% (9/15) of the elderly patients. The differences were not statistically significant (*p* = 0.883). This indicates that adult and elderly patients were just as likely to sustain complex elbow dislocations.

### Distribution of injury mechanism versus injury pattern

Analysing the distribution of injury mechanism versus injury pattern, 63% (19/30) of the patients with low-energy trauma sustained simple elbow dislocations, and 37% (11/30) complex elbow dislocations, respectively, 26% (11/42), and 74% (31/42) in cases of high-energy trauma. Thereby, the percentage of complex elbow dislocation increased significantly (*p* = 0.002) with the intensity of the injury mechanism, at the expense of simple elbow dislocations.

### Distribution of age versus mode of treatment

A total of 85% (61/72) of patients underwent surgery. Evaluating the relationship between age and mode of treatment, 84% (48/57) of the adult patients were treated operatively versus 87% (13/15) of the elderly patients. The difference was not statistically different (*p* = 1.000). Therefore, in the present study population, elderly patients were not less likely to get surgical treatment as compared to adult patients.

### Distribution of injury pattern versus mode of treatment

Reviewing the relationship between injury pattern and mode of treatment, simple elbow dislocations were treated conservatively in 27% (8/30) and with surgery in 73% (22/30) compared to 7% (3/42), and 93% (39/42) for complex elbow dislocations.

### Mode of surgical treatment

**Fig. 4:**
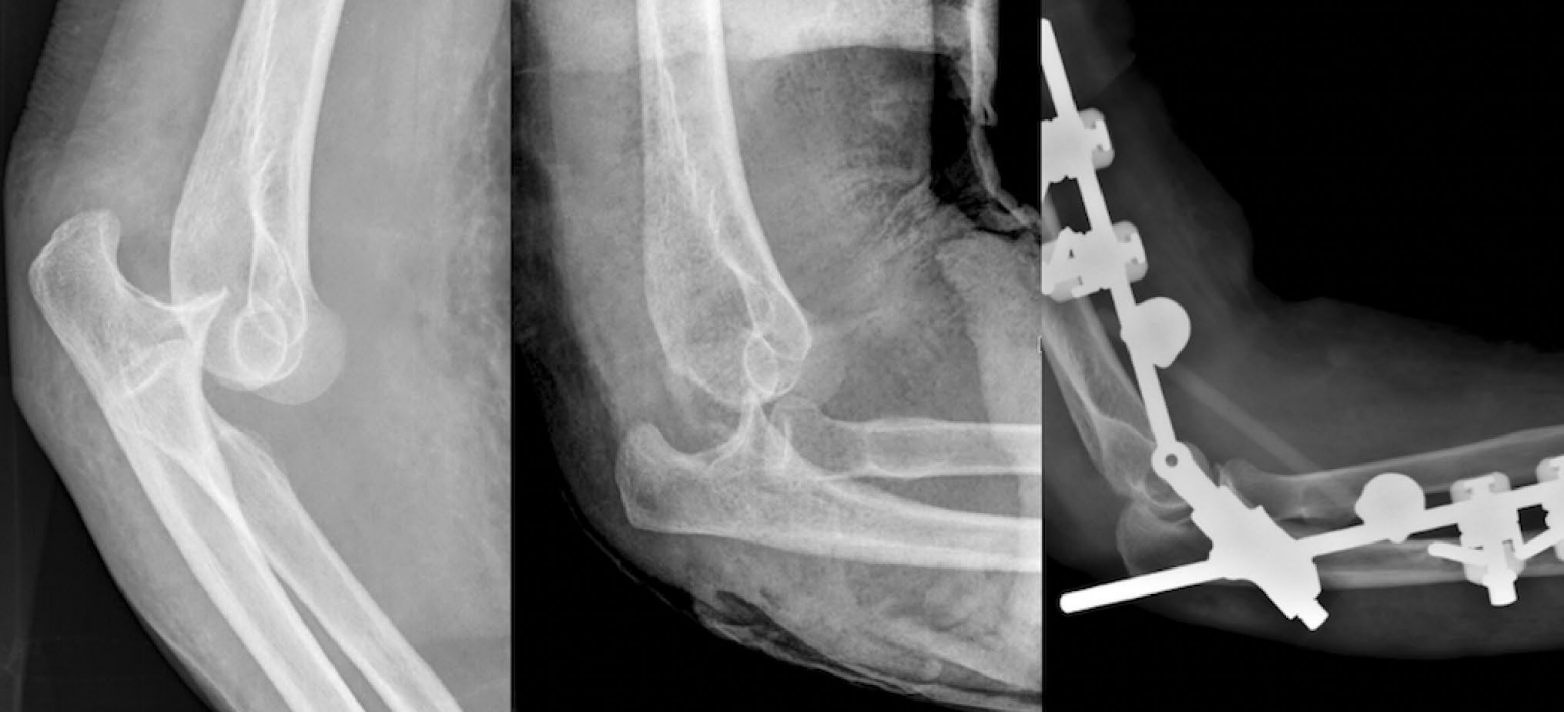
Lateral radiographs of unstable simple elbow dislocation. **a** Posterior elbow dislocation. **b** Subluxated joint after closed reduction. **c** Ligament repair with suture anchor and protective fixation with DJD hinged external fixator

For unstable simple elbow dislocations, ligament repair with suture anchor was performed in 45% (10/22) of both the medial and lateral collateral ligament, and isolated medial in 45% (10/22), and lateral in 9% (2/22). Protective fixation was used in 23% (5/22) with a DJD hinged external fixator in 60% (3/5), and a Kirschner wire in 40% (2/5) (Fig. 4, Table 3).

**Table 3:** Mode of surgical treatment in unstable simple elbow dislocations

Ligament repair	100% (22/22)
Medial/lateral collateral ligament	45% (10/22)
Medial collateral ligament	45% (10/22)
Lateral collateral ligament	9% (2/22)
Protective fixation	23% (5/22)
DJD hinged external fixator	60% (3/5)
Kirschner wire	40% (2/5)

**Fig. 5:**
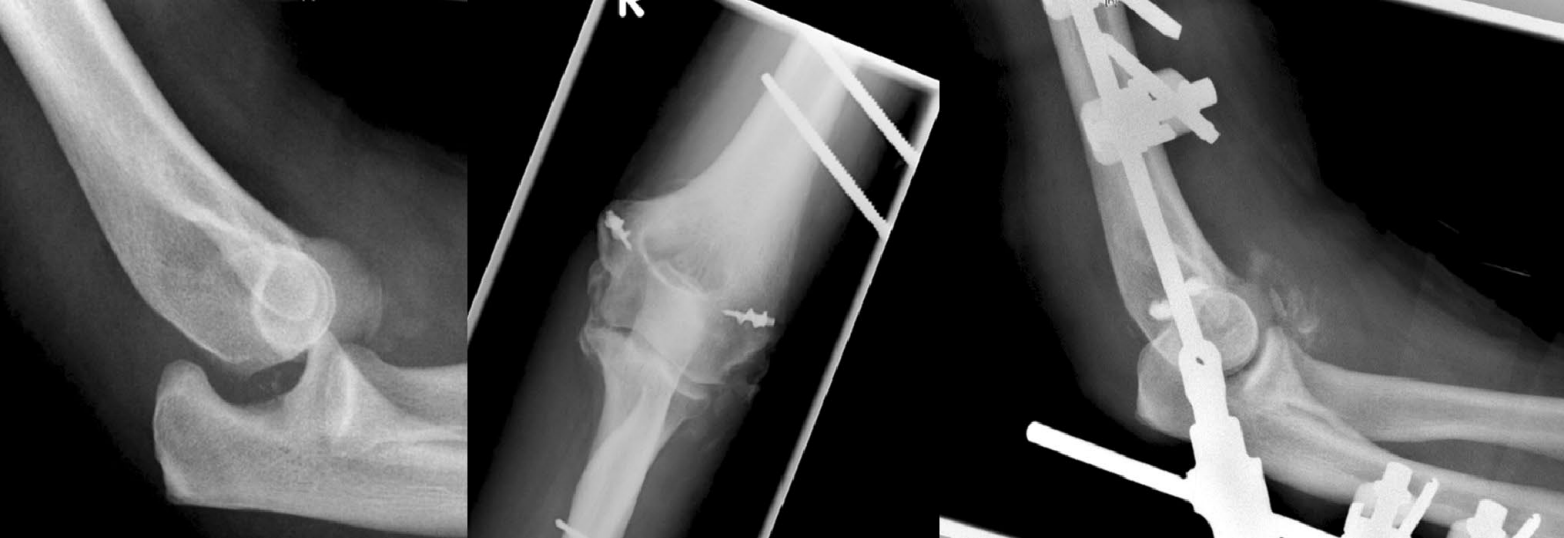
Antero-posterior and lateral radiographs of complex elbow dislocation. **a** Subluxated joint after closed reduction. **b**, **c** Ligament repair with suture anchors and protective fixation with DJD hinged external fixator

Concerning complex elbow dislocations, radial head fractures were treated surgically in 91% (29/32) of the cases, mainly with screws (41%, 12/29). Fractures of the ulnar coronoid process underwent surgery in 75% (21/28), primarily with transosseous suture (43%, 9/21). Olecranon fractures were managed operatively in 100% (10/10) with locking plates in 90% (9/10). Ligament repair with suture anchor occurred in 74% (29/39), while protective fixation was applied in 28% (11/39) (Fig. 5, Table 4).

**Table 4:** Mode of surgical treatment in complex elbow dislocations

Radial head	91% (29/32)
Screw	41% (12/29)
Locking plate	28% (8/29)
Radial head prosthesis	28% (8/29)
Radial head resection	3% (1/29)
Ulnar coronoid process	75% (21/28)
Transosseous suture	43% (9/21)
Screw	33% (7/21)
Suture anchor	14% (3/21)
Transosseous suture/locking plate	5% (1/21)
Locking plate	5% (1/21)
Olecranon fracture	100% (10/10)
Locking plate	90% (9/10)
Tension band wiring	10% (1/10)
Ligament repair	74% (29/39)
Medial/lateral collateral ligament	48% (14/29)
Lateral collateral ligament	41% (12/29)
Medial collateral ligament	10% (3/29)
Protective fixation	28% (11/39)
DJD hinged external fixator	91% (10/11)
Kirschner wire	9% (1/11)

## Discussion

Age and gender are both important factors for defining risk for sustaining acute elbow dislocations [4, 5, 8]. Previous studies have indicated a male predominance in the occurrence of elbow dislocations and a significantly higher risk for this injury for adult patients [8, 9]. The results of the present study were congruent showing predominantly male patients and a similar age profile.

Most elbow dislocations are the result of falls onto the outstretched arm [3, 6, 8, 10]. Our data were similar in elderly patients with 73% of the elbow dislocations occurring as the result of a fall. However, in the adult population, high-energy trauma was the most common cause of injury (67%).

Posterior dislocations have proven to be the most common direction of displacement by far and can be further subdivided into posterolateral and posteromedial, of which posterolateral accounts for over 80% [3, 11]. The results of the present study were consistent with 79% posterior dislocations, thereof 71% posterolateral.

Concerning injury pattern, the literature indicates that simple elbow dislocations are the most common type of injury, and up to 20% of dislocations are associated with fractures [3, 12]. Our data were different with a higher rate (58%) of complex elbow dislocations. This could be due, in part, to our higher proportion of high-energy trauma in the adult population.

Simple elbow dislocations are mainly treated with conservative management in current literature [3, 7, 13–15]. Many authors have reported favorable results after conservative treatment for simple elbow dislocations [10, 13, 16, 17]. Additionally, some studies have reported more satisfactory results after conservative treatment compared to surgical management [11, 18]. However, indication for surgical repair is generally recommended in unstable elbow dislocations, in which primary ligament repair demonstrated satisfactory outcomes [11, 17, 19–21]. Therefore, it is standardly indicated to determine the treatment method of simple elbow dislocations according to the stability after reduction of the elbow joint [7, 17]. If the elbow is radiological subluxated or non-congruent after reduction, unstable under anaesthesia, or requires an extension limitation over 30–45° to maintain reduction, unstable simple elbow dislocation has to be diagnosed, recommending early ligament repair [7]. Our data were consistent, but the percentage of patients with unstable simple elbow dislocations was higher compared to current data. In our study cohort, surgery had to be performed in 73% of the simple elbow dislocations due to remaining instability or non-congruency of the reduced elbow. Congruently, disruption of both the medial and lateral collateral ligament was detected in 45% of the cases compared to isolated medial in 45%, and lateral in 9%.

The treatment principles of complex elbow dislocations are mainly reduction of the joint, stabilisation of associated fractures, and early motion [2, 4, 12]. Ligament repair and hinged external fixators are necessary in some cases to restore stability for early motion [11, 12, 22–24]. The results of the present study were similar. Screws (41%) predominated the surgical treatment of the radial head compared to transosseous suture (43%) for the ulnar coronoid process, and locking plate (90%) for the olecranon. Ligament repair with suture anchor occurred in most complex elbow dislocations (74%), while protective fixation was applied in 28%.

Age has been shown to be a major determinant of non-operative management [25]. Contrary, in the current study, elderly patients were just as likely to get surgical treatment as adult patients due to a variety of reasons. First, the high percentage of complex elbow dislocations (60%). Second, it was also due to a personal request of the elderly patients who are now more active than ever and often prefer surgical treatments that do not hamper their activities.

Some limitations must be considered for the present study. First, the study design was retrospective. Second, our data provided no information on outcomes. Even though this study contributes to currently available epidemiological data, the definite answer regarding appropriate algorithm for treating acute elbow dislocations requires prospective long-term outcome studies.

## Conclusion

In conclusion, the treatment method of acute elbow dislocation has to be determined according precise injury pattern and instability after reduction of the elbow joint. To achieve a congruent and stable joint, we recommend primary surgical repair as first-line treatment for patients with unstable simple and complex elbow dislocation independent of age.

## Data Availability

Not applicable.
